# A simple model of wake capture aerodynamics

**DOI:** 10.1098/rsif.2023.0282

**Published:** 2023-09-27

**Authors:** Mostafa R. A. Nabawy

**Affiliations:** ^1^ School of Engineering, The University of Manchester, Manchester M13 9PL, UK; ^2^ Aerospace Engineering Department, Faculty of Engineering, Cairo University, Giza 12613, Egypt

**Keywords:** wake capture, wing–wake interaction, flapping wings, insect flight, aerodynamics, analytical modelling

## Abstract

Flapping wings may encounter or ‘capture’ the wake from previous half-stroke, leading to local changes in the instantaneous aerodynamic force on the wing at the start of each half-stroke. In this paper, I developed a simple approach to integrating prediction of these wake capture effects into existing analytical quasi-steady models for hovering insect flapping flight. The local wake flow field is modelled as an additional induced velocity component normal to the stroke plane of the flapping motion that is blended/switched in at the start of each half-stroke. Comparison of model results against experimental data in the literature shows satisfactory agreement in predicting the wake capture lift and drag variations for eight different test cases. Sensitivity analysis shows that the form of the translation velocity time history has a significant effect on the magnitude of wake capture forces. Profiles that retain high translational velocity right up to stroke reversal evoke a much larger effect from wake capture compared with sinusoidal. This result is significant because while constant flapping translation velocity profiles can be generated in the laboratory, the very high accelerations required near stroke reversals incur high mechanical cost that prevents practical adoption in nature or engineered flapping flight vehicles.

## 1. Introduction

**Figure 1.**
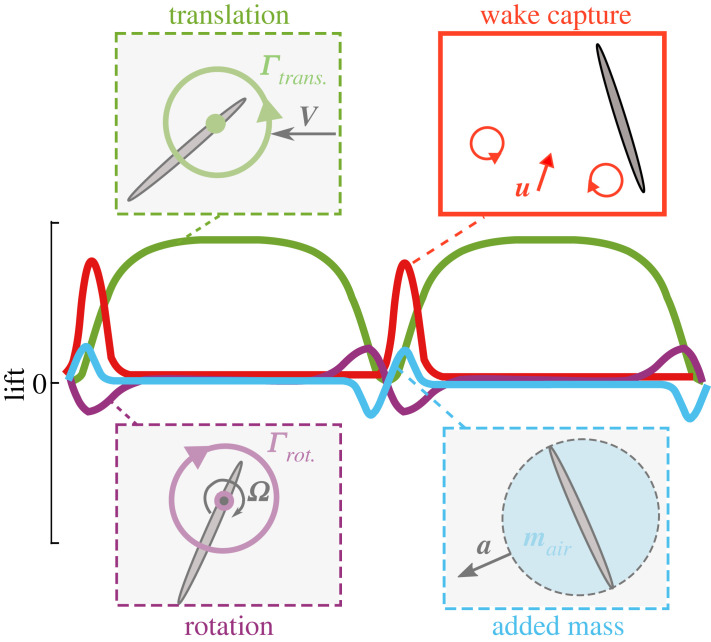
Qualitative description of lift time history for symmetric forward and backward half-strokes of a flapping insect wing in hover. Aerodynamic mechanisms are: translation (green), rotation (purple), added mass (light blue) and wake capture (red). In this example, a flapping wing translating with a velocity, ***V***, creates a circulation, $\Gamma_{trans.}$*.* At high angles of attack, an attached leading-edge vortex will form enhancing lift through prevention of stall. During stroke reversal, an additional circulation, $\Gamma_{rot.}$, is created due to the wing rotational velocity, Ω*.* The non-circulatory added mass effect contributes to force generation depending on the surface normal acceleration profile, ***a***, of the air mass, $m_{air}$, surrounding the wing. A contribution due to wake capture is illustrated based on presence of an induced velocity field, ***u***, from vortices of the wake from the previous half-stroke.

Flapping wing insect flight is rich in complex aerodynamic phenomena that are not yet adequately explained and at first glance appear beyond the capability of analytical modelling. The overall goal of this work is to develop improved understanding of a specific aerodynamic mechanism known as *wake capture* that can have an important effect at the start of each flapping half-stroke. For completeness, the *main* aerodynamic mechanisms contributing to force generation in hovering insect flight are illustrated in figure 1: (i) Wing translation provides the main circulation [1]. A leading-edge vortex (LEV) will form at high angles of attack, and this LEV tends to remain stably attached, preventing stall and augmenting lift beyond values that would be normally expected from classical fixed-wings [2–4]. (ii) Wing rotation at the end of each half-stroke provides additional circulation with a contribution that depends on whether the rotation timing is advanced, symmetric or delayed with respect to translation [2,5]. (iii) Acceleration and deceleration of the surrounding air closest to the wing generates an inertial force and is referred to as an ‘added mass’ effect [1,6]. (iv) Wake capture (also referred to as wing–wake interaction) occurs when the wing encounters or ‘captures’ the wake from previous half-stroke, leading to local changes in the instantaneous aerodynamic force at the start of each half-stroke [1,5]. While other aerodynamic mechanisms, e.g. clap-and-fling, can also influence the aerodynamics, their effect is more limited to specific insects, hence may be considered secondary compared with the previously discussed mechanisms.

Wake capture is less well understood compared with the other aerodynamic mechanisms of insect flight, and there have been relatively few experimental or numerical efforts dedicated to developing understanding in this area. The effect of wake capture within three-dimensional flapping wing aerodynamics started to attract serious attention after the work of Dickinson and his group almost two decades ago [7,8]. Conducting experiments on a dynamically scaled model of a fruit fly wing, they noticed an increase in the aerodynamic force generated at the start of each half-stroke. This could not be explained from rotational circulation alone, and hence was attributed to the interaction between the wing and its wake from the previous half-stroke. Despite these early insights, only a *handful* of subsequent studies have attempted to develop the concept further. For example, Lua *et al*. [9] considered a more fundamental experimental characterization of wake capture on a two-dimensional flapping wing, showing that either lift enhancement or reduction can happen depending on the flapping motion and timing of stroke reversal. Later the same group considered a three-dimensional fruit fly wing case with time-advanced wing pitching rotation kinematics [10]. Using both numerical and experimental methods they showed that modifying the duration of translation acceleration and deceleration has a significant effect on the aerodynamic force, suggesting that wing kinematics play a major role in defining the extent to which wake capture could be exploited. In a more recent study, numerical simulations were conducted for rectangular wings undergoing sinusoidal kinematics [11]. Differences between two-dimensional and three-dimensional effects on wake capture were explored by varying the wing aspect ratio and Rossby number, showing that three-dimensionality plays a role in controlling the efficacy of mechanisms underlying wake capture.

The physics behind wake capture has been a topic of vigorous scientific debate, with several conflicting views from the different research groups. This debate was started by a numerical study from Sun & Tang [12] calculating the unsteady aerodynamic forces on a fruit fly flapping wing using kinematics similar to the benchmark experiments by Dickinson *et al*. [7]. Numerical modelling did not adequately capture the aerodynamic force time histories at the beginning of half-strokes where wake capture effects should be present. While other numerical models (such as [13]) managed to show wake capture effects similar to those in the benchmark experimental results, an argument was cast by Sun and Tang that the increase in aerodynamics forces at the start of a half-stroke was due to the rapid acceleration of the wing [12]. This argument led to further investigations [14,15], comparing forces generated within the first and subsequent strokes for the fruit fly case. The main finding of these investigations was that the force peaks attributed to wake capture cannot be ignored but prediction is highly dependent on the details of the wing kinematics. This debate shows that there may be no unique physical explanation for what happens during stroke reversals. However, the currently available studies are relatively limited in scope and there is a real need for more detailed experimental data to be produced across a wider range of planform geometries, wing motions and flow conditions.

Wake capture is a local effect within the flapping cycle but can have potentially significant global effect on lift capability of a hovering insect. Forces due to wake capture depend on estimation of the transient aerodynamic response of the wing to the combined wing motion due to the flapping kinematics and the local induced velocity field in the wake. While modelling the aerodynamic response to kinematic variations is established, analytical function representations for the induced velocity field due to wake flows currently do not exist, probably due to a preconceived assumption that it must be too complicated. There is, therefore, a strong need to develop analytical models that allow *reasonable* prediction of wake capture aerodynamics and hence answer important outstanding questions such as: (i) What novel mathematical formulations can be used to best represent the induced velocity field driving the underlying physics of wake capture? (ii) What wing shapes and kinematics allow best exploitation of the wake capture effect? (iii) To what extent does the incorporation of wake capture within analytical models explain some insect flight behaviours and/or inform engineering design of flapping wing vehicles? While the problem of wake capture is challenging, given recent successes in establishing simple and computationally inexpensive models to describe other complex aerodynamic behaviours, e.g. [16–24], there are reasons to be optimistic that further sustained work on the wake capture problem will reveal similarly useful modelling insights. In fact, the aim of the model of wake capture developed in this work is to enable an improved understanding of the physics underlying wake capture, provide a prediction tool of its impact on aerodynamic performance and allow the identification of the key parameters that control its utility.

## 2. A simple model for a complex problem

### 2.1. Preliminaries

The hypothesis of this work is that globally important aspects of complex transient wing–wake interaction in hovering flapping flight can be successfully modelled using novel induced velocity field representations based on primitive geometric insights. The starting point is the expressions of the instantaneous lift and drag forces, *L* and *D*, on a hovering flapping wing

2.1 $$L(t)=\frac{1}{2}\;\rho V_{t}^{2}(t){\hat{r}}_{2}^{2}SC_{L}(t)$$

and

2.2 $$D(t)=\frac{1}{2}\;\rho V_{t}^{2}(t){\hat{r}}_{2}^{2}SC_{D}(t),$$

where *ρ* is the fluid density, *V_t_* is the wing tip translation velocity, ${\hat{r}}_{2}\;$is the non-dimensional radius of the second moment of wing area [25], *S* is the wing area and *t* denotes time. The lift and drag coefficients (*C_L_* and *C_D_*) are represented here using the well-established relations typically employed within insect-like flapping translation [26]

2.3 $$C_{L}(t)=C_{L\alpha }(t)\sin(\alpha (t))\cos(\alpha (t))$$

and

2.4 $$C_{D}(t)=C_{D0}+C_{L}(t)\;\tan(\alpha (t)),$$

where, *α* is the angle of attack, *C*_*Lα*_ is the three-dimensional wing lift curve slope, and *C*_*D*0_ is the wing drag coefficient at zero-lift. Note that, in equation (2.3), the lift curve slope is written as a function in time, as the current model captures the transient wake capture aerodynamics within the three-dimensional wing lift curve slope expression. This approach is consistent with the recent study of Hu *et al*. [27], who found, using computational fluid dynamics (CFD) simulations, that lift and drag coefficients due to wake capture follow the exact same angle of attack trigonometric variations as that of the translation (absence of stall) aerodynamic mechanism but with a much-damped amplitude. Hence, the wake capture effect was accounted for via tuning the amplitudes of the same trigonometric functions used to represent the translational aerodynamics, rather than changing the mathematical forms of the functions.

An expression for the lift curve slope is obtainable following the basic aerodynamic approach, starting with the expression of the lift coefficient of a wing with a symmetric section in the vicinity of small angles of attack

2.5 $$C_{L}=C_{l\alpha ,2d}(\alpha (t)-\alpha_{i}(t)),$$

where *C_lα_*_,2*d*_ is the two-dimensional section lift curve slope, and in this work is assigned a value of 0.09 deg^−1^ based on experimental flat plate lift curve slope values at typical insect Reynolds numbers [28,29]. The symbol *α_i_* denotes the induced angle of attack resulting from the induced downwash velocity and is function of time, as it is through this term the model will capture the transient variations of the induced angle of attack due to the wake capture velocity field. It is important to stress that while the wake capture is in reality an induced velocity effect, it is represented here as an induced angle of attack instead. This is to ensure model simplicity, and for consistency with the basic aerodynamic approach typically employed for obtaining an expression for the three-dimensional wing lift curve slope. Note that, the minus sign preceding the induced angle of attack term in equation (2.5) is to follow the typical notation within the classical aerodynamic literature, e.g. [30]. However, in the context of this study, the induced angle of attack can be positive or negative to account for the fact that wing–wake interaction can either increase or decrease the effective angle of attack (and hence the aerodynamic force) depending on the developed vortex structures. That said, in the current study, only the favourable case of enhancing the effective angle of attack will be considered. Finally, a proposed general representation for the induced angle of attack is expressed as

2.6 $$\alpha_{i}(t)=-A\times S(t),$$

where *A* is the amplitude of the induced angle of attack and *S*(*t*) is a switching function term to control the unsteady nature of the wake capture effect. Briefly, this switching function is a modulating function that changes value from + 1 to − 1 in relation to the control variable value relative to some reference point. Switching functions are typically used within aerodynamics to represent transition from one domain to another. They were first presented by Hewes [31] to model switching from the linear to the nonlinear lift coefficient curve regions for a three-dimensional wing. In the context of this work, *S*(*t*) = +1 indicates the start of influence of wake capture, whereas *S*(*t*) = −1 indicates end of influence of wake capture (or absence of wake capture effect). Hence, in the absence of wake capture, the notations used in equations (2.5) and (2.6) lead to a decrease in the effective angle of attack, as expected.

### 2.2. Representation of the induced angle of attack

To evaluate the time variation of the induced angle of attack, both *A* and *S*(*t*) should be determined. Both are understood to rely on the strength and motion of the vortex structures encountered by the wing. However, here I adopt a *simplified* approach by setting the value of *A* and selecting a suitable function representation for *S*(*t*) that would allow a best fit/representation of the available experimental data. To obtain a set value of *A*, I start by making the reasonable assumption that the amplitude of the induced angle of attack from a local wake interaction is equal to the well-known expression of *α_i_*_,ref_ written as

2.7 $$\alpha_{i,\mathrm{ref}}=\frac{kC_{L}}{\pi AR}=\frac{C_{L}}{\pi AR_{\mathrm{eff}}},$$

where *C_L_* is the three-dimensional wing lift coefficient, *AR* is the wing geometric aspect ratio, and *k* is the induced power factor [19]. Note that *AR*_eff_ is the well-known ‘effective’ aspect ratio, defined as the ratio of the geometric aspect ratio to the induced power factor. Once *A* is set to a selected reference, then the switching function job is to control the variation of the induced angle of attack from the start of the half-stroke (when wake capture effect kicks-in, *S*(*t*) = +1) till it eventually reaches to the expected steady-state value (*S*(*t*) = −1) at a certain non-dimensional time of the flapping cycle denoted here as (*t*/*T*)_cut_, where *T* is the flapping period. In other words, (*t*/*T*)_cut_ represents the time within the flapping cycle after which the wake capture effect vanishes and the induced angle of attack value reaches its steady-state value.

Before proceeding any further, there are two points to highlight here. Firstly, the modelling approach presented here starts with making the sensible assumption of setting *A*, and then working out the switching function shape. However, if a convenient switching function—capable of capturing the available experimental data—is not reached, then a new setting for *A* should be proposed. Secondly, the reference value (*t*/*T*)_cut_ will be assumed here based on existing experimental and numerical data. Fortunately enough, the available experimental and computational data to date [7–11,13,14] shows that the transient wake capture force starts developing immediately after the end of the previous half-stroke, reaching a peak, and then diminishing entirely at a duration of about 20% of the half-stroke, i.e. (*t*/*T*)_cut_ of 0.1 (more discussions on this value will be provided in §4.2). Now, combining equations (2.5), (2.6) and (2.7), an expression for lift curve slope is obtained as

2.8 $$C_{L\alpha }(t)=\frac{C_{l\alpha ,2d}}{1-(\left(C_{l\alpha ,2d\;}/(\pi AR_{\mathrm{eff}}))\;S(t\right))}.$$

Note that, in the absence of wake capture effects, the switching function term, *S*(*t*), would tend to −1; hence reverting to the classical lifting line theory expression for a three-dimensional wing lift curve slope.

### 2.3. Time variation of the switching function

**Figure 2.**
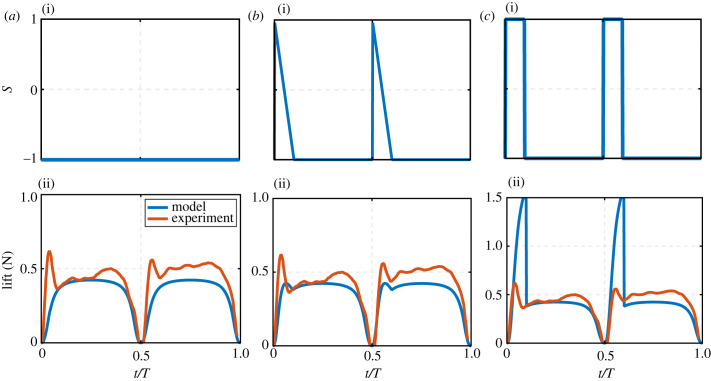
Comparison of the model performance for three different switching functions: (*a*) No switching, (*b*) linear switching, and (*c*) step switching. (i) Variation of the switching function throughout the flapping cycle. (ii) Comparison of the model results against the experimental lift force results sourced from Sane & Dickinson [8]. Experimental results are for symmetric half-strokes. Experimental lift values include the translation and wake capture effects only, i.e. rotational circulation and added mass components are subtracted from the total lift values.

A sensible starting point is to investigate the case where wake capture is absent, and this case can be represented by setting *S*(*t*) throughout the flapping cycle to the constant value of − 1, figure 2*a*(i). In doing this, only the steady-state translation aerodynamics of the flapping half-stroke is simulated, with no consideration of the wake capture transients, figure 2*a*(ii). Next, two intuitive selections of the switching function were investigated to assess their performance in capturing the wake capture peaks from the experiments of Sane & Dickinson [8], used here as the benchmark test case. The first of these functions was a linear variation, figure 2*b*(i), represented as

2.9 $$S=\{\begin{array}{cc} 1-2\frac{\left(t/T\right)}{{\left(t/T\right)}_{\mathrm{cut}}} & 0\leq (t/T)\leq {\left(t/T\right)}_{\mathrm{cut}}\\ 1-2\frac{(t/T)-0.5}{{\left(t/T\right)}_{\mathrm{cut}}} & 0.5\leq (t/T)\leq 0.5+{\left(t/T\right)}_{\mathrm{cut}}\\ -1 & \mathrm{otherwise} \end{array}.$$

The second switching function assessed was a step variation, figure 2*c*(i), representing the upper bound for all possible switching variations

2.10 $$S=\{\begin{array}{cc} 1 & 0\leq (t/T)\leq {\left(t/T\right)}_{\mathrm{cut}}\\ 1 & 0.5\leq (t/T)\leq 0.5+{\left(t/T\right)}_{\mathrm{cut}}\\ -1 & \mathrm{otherwise} \end{array}.$$

The performance of these two functions in predicting the lift variation of the fruit fly experimental results of Sane & Dickinson [8] is shown in figure 2*b*(ii) and *c*(ii), respectively. The main outcome of this exercise is that the linear variation is not sufficient to produce peak amplitudes similar to those of the experimental results, whereas the step variation produces peaks that are much higher in amplitude than the experimental ones. This result has two implications: first, the value of *α_i_*_,ref_ hypothesized for the amplitude *A* is sufficient and there is no need to change the amplitude further. Second, a smoother variation of the switching function that falls between the linear and the step variations is required to conveniently represent the wake capture variation.

Based on the aforementioned requirements, an exponential formulation for the switching function was conceived and expressed as

**Figure 3.**
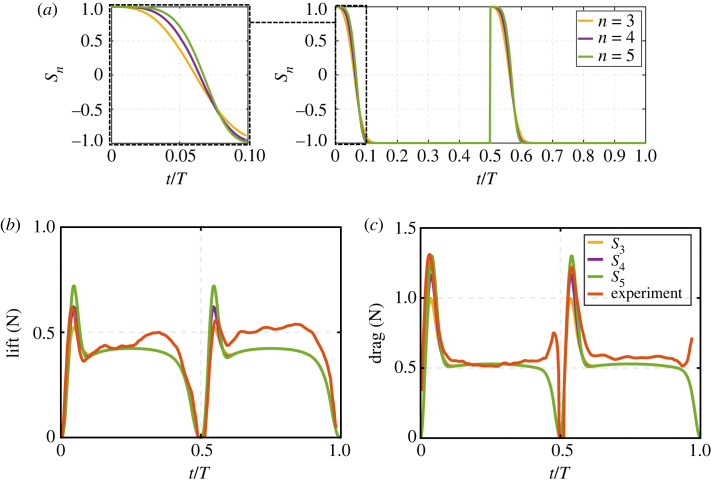
Comparison of the model performance with exponential switching for different switching gains, *n*, against lift and drag experimental results from Sane & Dickinson [8]. It could be seen that *n* = 4 provides a convenient matching between the model and experimental data. Experimental results are for symmetric half-strokes. Experimental force values include the translation and wake capture effects only.

2.11 $$S_{n}(t)=\{\begin{array}{cc} 2e^{-n{\left(\;(t/T)/{\left(t/T\right)}_{\mathrm{cut}}\right)}^{n}}-1 & (t/T)\leq 0.5\\ 2e^{-n{\left(\;((t/T)-0.5)/{\left(t/T\right)}_{\mathrm{cut}}\right)}^{n}}-1 & 0.5<(t/T)\leq 1 \end{array},$$

where *n* is a switching gain playing a significant role in the way the function behaves with respect to the control variable (*t*/*T*). Note that the exponential switching function now takes a subscript *n* for referencing purposes. Once the switching gain is decided, equation (2.11) is used in conjunction with equations (2.3), (2.4) and (2.8) to model the aerodynamic coefficients. Figure 3 shows the model performance with exponential switching against the experimental results of Sane & Dickinson [8] for different values of *n*. It is evident that exponential switching is conveniently able to capture the experimental results through selecting a suitable switching function gain, *n*, to best represent the experimental data. For example, for the fruit fly experimental results of Sane & Dickinson [8], selecting *n* of four allows for a good match of the time history of the model lift and drag variations, figure 3. Note that the input parameters for the demonstration shown in figure 3 will be comprehensively explained in §3.1.

## 3. Results

### 3.1. Comparison against experimental results of fruit fly and hawkmoth

**Figure 4.**
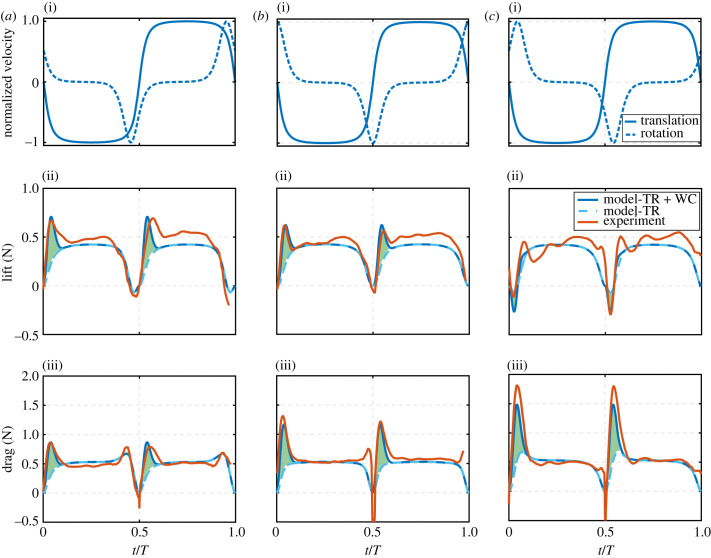
Comparison of model output against experimental data for (*a*) advanced pitching rotation, (*b*) symmetric pitching rotation and (*c*) delayed pitching rotation. (i) Shows wing kinematic variations, (ii) shows lift forces, and (iii) shows drag forces. Both translation and rotation velocities are normalized by respective maximum velocity values to better compare/visualize the waveform variations. Maximum translation velocity was 0.3 m s^−1^ and maximum rotation velocity was calculated to allow a 45° angle of attack at mid-half-strokes. TR denotes translation effect, whereas WC denotes wake capture effect. Dark blue solid lines represent model results including both translation and wake capture effects. Light blue dashed lines represent model results for translation effect only, and hence the difference between these two lines represents the wake capture contribution (green shading). Experimental data are sourced from Sane & Dickinson [8].

In this section, the proposed model will first be compared against available experimental results from the literature. Note that experimental studies considered for comparison here are those that provide sufficient information on the conducted experiments (including wing morphology, kinematics and testing conditions) to allow the necessary inputs to run the model. The model is first compared against the experimental results from Sane & Dickinson [8], using the exact same set-up employed in their experiment. The fluid density is set to that of mineral oil. Wing geometric and aerodynamic parameters employed are provided in table 1. These parameters are substituted into the model while using a switching gain of four. Results are compared against the experimental results in figure 4 where the lift and drag forces are compared for all three wing pitching rotation cases (advanced, symmetric and delayed). Note that the force values shown in figure 4 include the effects of wing translation and wake capture only, i.e. the experimental force values in figure 4 are obtained by subtracting the rotational circulation and added mass force components from the measured total force values. This follows the ‘wake capture isolation’ approach proposed by Sane & Dickinson [8] where wake capture is exposed by subtracting the sum of the translational, rotational and added mass force components from the total measured aerodynamic force. Despite its simplicity, the ability of the model in capturing the force variations for the three different kinematic cases, particularly the wake capture peaks, is remarkable.

**Table 1.** Values of the geometric and aerodynamic parameters used in the comparison process based on the experimental set-ups of Sane & Dickinson [8] and Han *et al*. [32]*.*

wing geometric parameters			
parameter	description	fruit fly value	hawkmoth value
*R*	wing length (m)	0.25 [8]	0.25^a^ [32]
*AR*	wing aspect ratio	3.73 [7,8]	3.09 [32]
${\hat{r}}_{2}$	non-dimensional radius of the second moment of wing area	0.632 [7,8]	0.525 [33]

aerodynamic parameters			
parameter	description	fruit fly value	hawkmoth value
*k*	induced power factor	1.4 [19]	1.3 [19]
*C_D_*_0_	Drag coefficient at zero-lift	0.4 [7,8]	0 [34]

^a^An additional wing offset needs to be taken into consideration.

Figure 4 confirms how wing pitching rotation plays a vital role in determining the extent to which wake capture could be exploited. For the advanced pitching rotation case, wake capture leads to an increase in both the average lift and drag forces by 14% and 11%, respectively. For the symmetric case the increase is 11% and 15% for lift and drag, respectively. As for the delayed case, there is a decrease in lift by 1% whereas there is an increase in drag by 18%. Hence, advanced pitching rotation has the best wake capture lift gain followed by symmetric, whereas a delayed wing pitching rotation is unfavourable from a wake capture lift point of view. Similarly for the drag results, advanced pitching leads to the least excessive drag due to wake capture followed by symmetric and then delayed. Hence, from a wake capture perspective, delayed pitching kinematics should be avoided as it decreases lift while drastically increasing drag.

**Figure 5.**
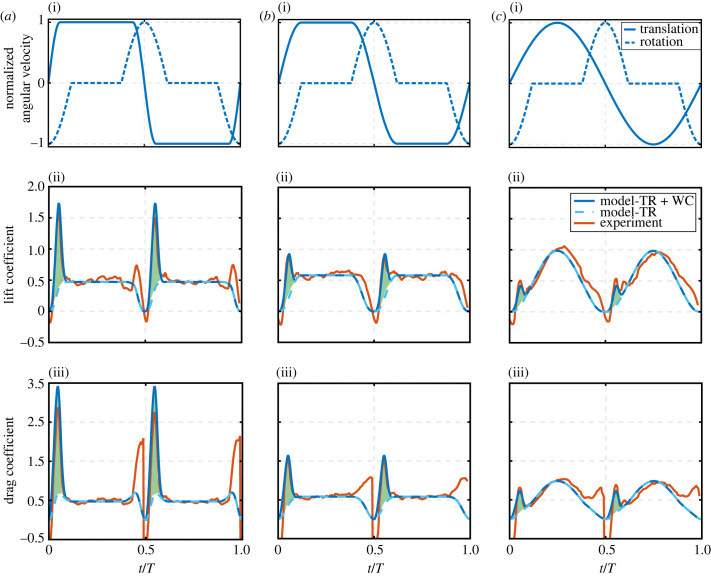
Comparison of model output against experimental data for different translation velocity profiles: (*a*) constant translation velocity over 76% of half-stroke, (*b*) constant translation velocity over 52% of half-stroke and (*c*) sinusoidal translation velocity. For all cases, a constant rotation velocity over 52% of half-stroke is employed. (i) Shows wing kinematic variations, (ii) shows lift coefficients, and (iii) shows drag coefficients. Both translation and rotation angular velocities of each test case are normalized by respective maximum velocity values to better compare/visualize the waveform variations. Maximum translation angular velocities were calculated based on a peak-to-peak flapping angle difference of 120°, and maximum rotation angular velocity was calculated to allow a 45° angle of attack at mid-half-strokes. TR denotes translation effect, whereas WC denotes wake capture effect. Dark blue solid lines represent model results including both translation and wake capture effects. Light blue dashed lines represent model results for translation effect only, and hence the difference between these two lines represents the wake capture contribution (green shading). Experimental data are sourced from Han *et al*. [32].

**Figure 6.**
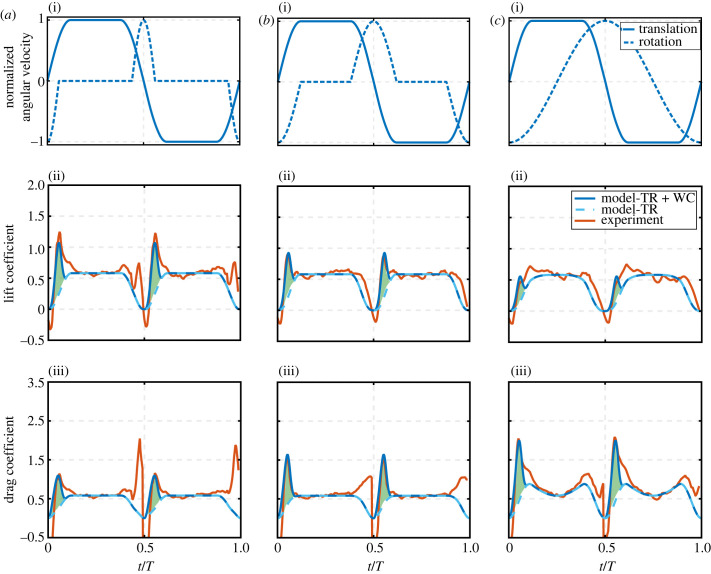
Comparison of model output against experimental data for different rotation velocity profiles: (*a*) 76% of half-stroke experiences constant rotation velocity, (*b*) 52% of half-stroke experiences constant rotation velocity, and (*c*) sinusoidal rotation velocity. For all cases, 52% of half-stroke experiences constant translation velocity. (i) Shows wing kinematic variations, (ii) shows lift coefficients, and (iii) shows drag coefficients. Both translation and rotation angular velocities of each case are normalized by respective maximum velocity values to better compare/visualize the waveform variations. Maximum translation angular velocity was calculated based on a flapping angle peak-to-peak difference of 120° and maximum rotation angular velocity was calculated to allow a 45° angle of attack at mid-half-strokes. TR denotes translation effect, whereas WC denotes wake capture effect. Dark blue solid lines represent model results including both translation and wake capture effects. Light blue dashed lines represent model results for translation effect only, and hence the difference between these two lines represents the wake capture contribution (green shading). Experimental data are sourced from Han *et al*. [32].

The second series of comparison cases presented herein will be against the experimental results from Han *et al*. [32] for their hawkmoth wing model experiments. In these experiments, their main objective was to provide lift and drag force measurements for various kinematic variations of the flapping angle and angle of attack for symmetric half-strokes. The same set-up employed in their experiment is used here: wing geometric and aerodynamic parameters from the experiment are provided in table 1. These parameters are substituted into the model with a switching gain of eight and results are compared against the experimental results in figures 5 and 6. Again, the lift and drag values shown in figures 5 and 6 only include the effects of wing translation and wake capture, i.e. the experimental force values in figures 5 and 6 are obtained by subtracting the rotational circulation and added mass force components from the measured total force values. Note that, for consistency with Han *et al*. [32], the results in figures 5 and 6 are shown in the form of coefficients where the aerodynamic forces were non-dimensionalized by $0.5\rho V_{\mathrm{ref}}^{2}S$, following definitions in [32].

There are three outputs that can be extracted from this comparison. First, from both figures, it could be seen that the model succeeds again in conveniently capturing the wake capture peaks for the different kinematics cases presented (the kinematics represents variations of both the velocity waveform (figure 5) and angle of attack waveform (figure 6)). The only concern may be that the model in these figures employed a different switching gain as compared with the previous fruit fly case demonstrated in figure 4; however, this issue will be discussed in detail in §4.2. Second, it is evident from figure 5 that as the velocity waveform retains a constant value for a longer period throughout the half-stroke, i.e. shorter acceleration and deceleration phases within the half-stroke, wake capture peaks become more evident. On the other hand, as the velocity waveform tends towards the sinusoidal variation, the wake capture peaks are much weaker. Third, figure 6 shows that when the angle of attack retains a constant value for a longer period within the half-stroke, i.e. smaller wing rotation phases, wake capture lift becomes more pronounced; however, as the angle of attack profile approaches a sinusoidal variation, wake capture lift is weaker. These observations will be confirmed in the discussion of the outputs within the next section (§3.2).

### 3.2. Sensitivity to input parameters

**Figure 7.**
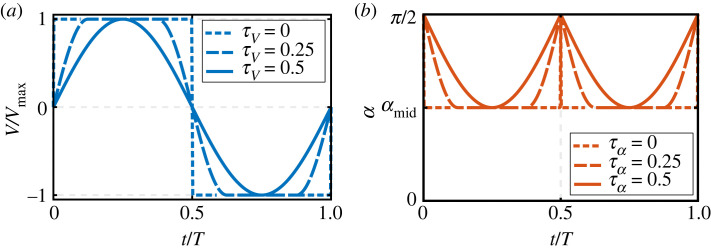
Kinematics waveform variations employed in the sensitivity analysis. (*a*) Translation velocity variations. (*b*) Angle of attack variations. In both cases, *τ* varies from zero (constant value) to 0.5 (sinusoidal waveform). *α*_mid_ represents the mid-half-stroke angle of attack.

Following the satisfactory comparison of the model results against available experimental measurements, it is instructive to understand how varying the model input parameters will affect the obtained results. However, to be able to conduct such a sensitivity analysis, it is important to represent the flapping kinematics in a suitable form for such analysis. Here, I will start the analysis by considering symmetric half-strokes for three reasons: (i) simplicity; (ii) symmetric half-strokes have been shown to well-expose wake capture effects, as opposed to delayed pitching rotation kinematics [8]; and (iii) symmetric half-strokes produce a zero net force from rotation circulation and added mass effects; hence, only translation and wake capture effects are expected to contribute to the *net* aerodynamic force production. That said, the effect of advanced wing rotation will also be considered later in this section. Figure 7 shows the kinematics representation of the translation velocity (to reach the same maximum translation velocity) and angle of attack (to reach the same mid-half-stroke angle of attack) waveforms for the symmetric half-strokes case employed in this study. Briefly, each waveform is composed of a constant motion at mid-half-stroke and a half-period sinusoidal function to connect the constant motions. The total non-dimensional acceleration-deceleration time controlling the velocity waveform is denoted by *τ_V_*, whereas the total non-dimentional rotation time controlling the angle of attack variation is denoted by *τ_α_*. In both cases, *τ* varies between 0 and 0.5; so that, if *τ* is zero, a constant velocity/angle of attack value along a half-stroke is simulated, whereas if *τ* is 0.5, a sinusoidal velocity/angle of attack waveform is obtained.

**Figure 8.**
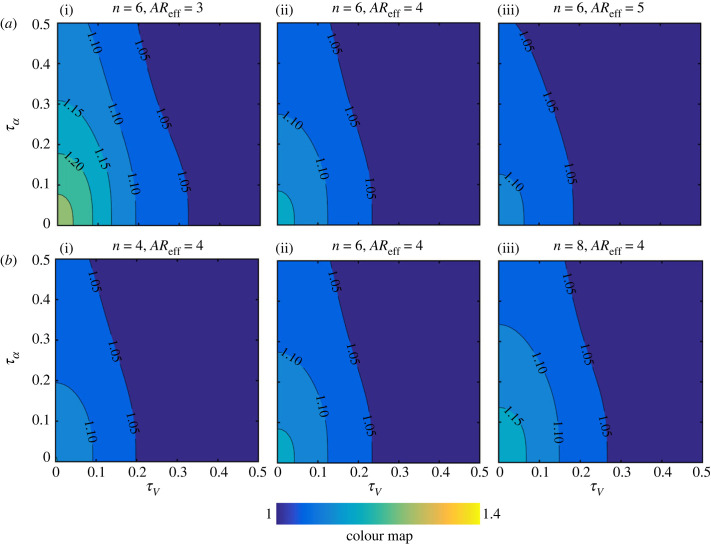
Contour plots for the ratio of the average lift force including wake capture and translation effects to average lift force including translation effect only ($\;{\bar{L}}_{TR+WC}/{\bar{L}}_{TR}$) for different *τ_V_* and *τ_α_* values. Results are for the symmetric rotation case. Problem input parameters are then varied within their typical values: (*a*) Effect of changing the effective wing aspect ratio. (*b*) Effect of changing the switching gain value.

**Figure 9.**
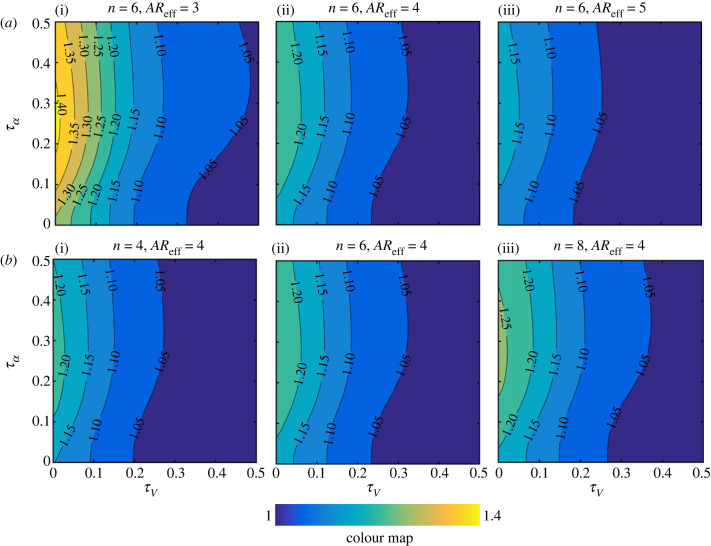
Contour plots for the ratio of the average drag including wake capture and translation effects to the average drag including translation effect only $\left(\;{\bar{D}}_{TR+WC}/{\bar{D}}_{TR}\right)$ for different *τ_V_* and *τ_α_* values. Results are for the symmetric rotation case. Problem input parameters are then varied within their typical values: (*a*) Effect of changing the effective wing aspect ratio. (*b*) Effect of changing the switching gain value. In this demonstration, the value of the drag coefficient at zero-lift is set to zero.

As a convenient representation of the kinematics variations has now been employed, the sensitivity analysis could now be set up. The outputs of this sensitivity analysis are the ratios for the average force including wake capture and translation effects to average force including translation effect only, i.e. $\;{\bar{L}}_{TR+WC}/{\bar{L}}_{TR}$ and $\;{\bar{D}}_{TR+WC}/{\bar{D}}_{TR}$. This way the additional contribution of wake capture, on top of the primary translation effect, can be easily assessed. On the other hand, the main two inputs investigated herein are the translation velocity waveform control parameter, *τ_V_*, and the angle of attack waveform control parameter, *τ*_α_. Then, other inputs, including the effective aspect ratio (*AR*_eff_) and switching gain (*n*), are varied once at a time within their typical values to ascertain their influence (figures 8 and 9).

Figure 8 provides the lift sensitivity results, demonstrating the *important outcome* that wake capture is largely tied to the shape of the velocity and angle of attack waveforms. Clearly, for all effective aspect ratios and switching gains, wake capture could not be of significant practical benefit unless the velocity approaches a constant value within half-strokes, i.e. minimum acceleration and deceleration durations. Similarly, for all cases shown in figure 8, it is evident that wake capture is maximally exploited when the angle of attack tends towards a constant value within half-strokes, i.e. minimum rotation durations. On the other hand, wake capture becomes of significantly less influence when *τ_V_* and *τ*_α_ approach 0.5, i.e. sinusoidal velocity and angle of attack waveforms within half-strokes. It is important to reiterate here that the variation trend of the contours shown in figure 8 is very similar for the different cases presented. This means that changing the effective aspect ratio and/or switching gain will not change the conclusion that wake capture becomes most beneficial when both velocity and angle of attack tend to be constant throughout half-strokes.

It should be noted that the three inputs capable of influencing the ratio $\;{\bar{L}}_{TR+WC}/{\bar{L}}_{TR}$ are the mid-half-stroke angle of attack, *α*_mid_, the wing effective aspect ratio, *AR*_eff_, and the switching gain, *n*. However, here, the mid-half-stroke angle of attack was fixed to a value of 45° known to deliver the maximum lift coefficient (see equation (2.3)). Figure 8*a* shows the influence of varying the effective aspect ratio. It could be seen that, as effective aspect ratio decreases, wake capture becomes more pronounced. This may seem unusual as it is well established within translational aerodynamics that decreasing *AR*_eff_ decreases lift coefficient. However, the opposite is happening here for the simple reason that wake capture is effectively modelled as an upwash effect rather than a downwash effect; hence, the flip of influence is not unexpected. Finally, figure 8*b* shows the influence of the switching gain on lift characteristics, where it is evident that as *n* increases wake capture produces more lift gain as expected (more discussions on the influence of switching gain will follow in §4.2).

I believe it is useful to stop here and consider the aspect ratio effect on wake capture lift, in light of some of the recent findings within the flapping wing aerodynamics field [35,36]. In particular, a recent experimental campaign from Addo-Akoto *et al*. [35] considered the wing aspect ratio influence on wake capture aerodynamics. The main outcome of this study was that lower aspect ratio values lead to a higher wake capture lift, which in turn can somehow compensate for the lower lift production of lower aspect ratio wings within the wing translation phase after wake capture has diminished. This result is consistent with the observed aspect ratio effect from the model presented herein. However, it is important to note that the conclusions from Addo-Akoto *et al*. [35] were based on an experimental investigation over an aspect ratio range between 2 and 4. This implies that the wings were not subject to strong tip stall effects. Note that recent experimental and numerical studies [37–39] have confirmed that wing tip stall occurs for revolving/flapping wings at radial wing locations greater than 3.5–5 times the mean geometric chord (measured from the centre of rotation). This takes me to a recent numerical study by Li and Nabawy [36], which conducted a comprehensive numerical analysis on wake capture aerodynamics of two-dimensional wings. The main outcome of this study was showing that the LEV state has a major influence on wake capture force production, with detached LEVs being able to produce beneficial wake capture lift, whereas attached LEVs being not equally able to produce the same positive wake capture lift effects. This implies that wings with relatively high aspect ratios (e.g. greater than 5) can exhibit some sort of wake capture lift enhancement from the detached LEV structures appearing near the wing tip. The aerodynamic picture that the current model is based on is that the wing does not experience stall. As such, the current model will not properly capture such wake capture effects of high aspect ratio wings. That said, it is known that most insect wing aspect ratios are clustered around 3–4 [25,40,41], for which the current model is expected to be fit for purpose. More importantly, hopefully this brief discussion has shed some light on the need for more future experimental and numerical studies to investigate the wake capture aerodynamics over a wide range of aspect ratios to enable a deeper understanding of the exact role that aspect ratio plays within wake capture aerodynamics.

**Figure 10.**
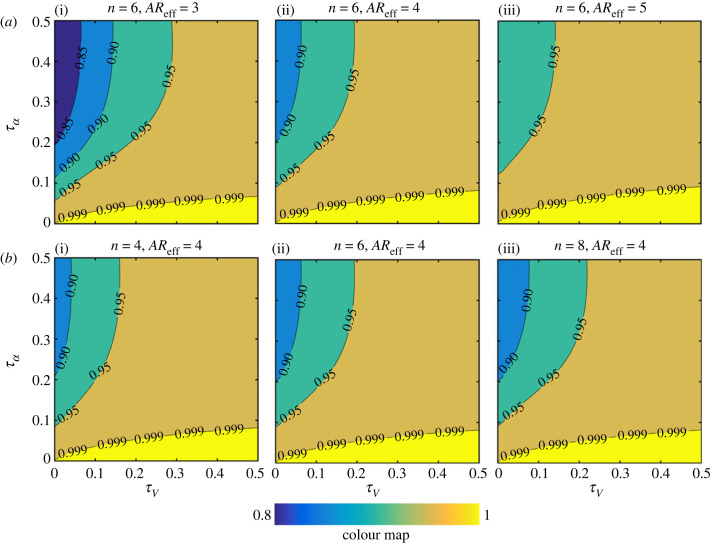
Contour plots for the lift gain-to-drag penalty ratio $\left(\left({\bar{L}}_{TR+WC}/{\bar{L}}_{TR})/({\bar{D}}_{TR+WC}/{\bar{D}}_{TR}\right)\right)$ for different *τ_V_* and *τ_α_* values. Results are for the symmetric rotation case. Problem input parameters are then varied within their typical values. (*a*) Effect of changing the effective wing aspect ratio. (*b*) Effect of changing the switching gain value. In this demonstration, the value of the drag coefficient at zero-lift is set to zero.

Figure 9 provides the drag sensitivity results for the symmetric half-stokes kinematics case. Clearly, it could be seen that contour values are always above unity. Hence while wake capture can provide extra lift it also comes with the penalty of more drag. Moreover, the contour values are generally higher than that of lift; hence, for symmetric half-strokes kinematics, the wake capture drag disadvantage is higher than wake capture lift advantage (see also figure 10). The way that the wing effective aspect ratio and switching gain influence the ratio $\;{\bar{D}}_{TR+WC}/{\bar{D}}_{TR}$ is similar to the lift sensitivity analysis. This is expected given that the drag coefficient is function of the lift coefficient (see equation (2.4)). Note that drag values are also influenced by the value of the drag coefficient at zero-lift, *C*_*D*0_; however, this has a much weaker effect on the variations presented and therefore is not considered here.

Another insightful observation from figure 9 is the fact that the contour lines show more reliance on *τ_V_*. In other words, the variation of the angle of attack is less important from a drag perspective, and the velocity profile carries the main responsibility towards the obtained drag ratio values. Clearly, as the velocity profile leads to having shorter acceleration and deceleration durations within half-strokes (*τ_V_* approaches 0), the wake capture drag disadvantage increases. Given that the drag contours are different in form compared with the lift contours, it is therefore instructive to assess the *lift gain-to-drag penalty* ratio obtained from the proposed model, which is conceived here as a measure of the wake capture aerodynamic efficiency. Figure 10 shows the contours of this ratio $\left(\left({\bar{L}}_{TR+WC}/{\bar{L}}_{TR})/({\bar{D}}_{TR+WC}/{\bar{D}}_{TR}\right)\right)$ for the different input parameters values. It is evident that this ratio is strongly dependent on the kinematic waveforms but is less sensitive to the effective aspect ratio and switching gain values. In particular, there are two main outcomes from this demonstration; firstly, the lift gain-to-drag penalty ratio is always below unity for symmetric half-strokes, and secondly, a unity value of this ratio can be achieved only if the angle of attack retains a constant value through half-strokes. In other words, for symmetric half-strokes the *drag penalty* will always be higher than *lift gain*, and in the best-case scenario (i.e. constant angle of attack) they will be equal. Additionally, it could be seen that as the contours approaches unity, the effect of *τ_V_* becomes absent, i.e. the velocity kinematics waveform does not matter.

**Figure 11.**
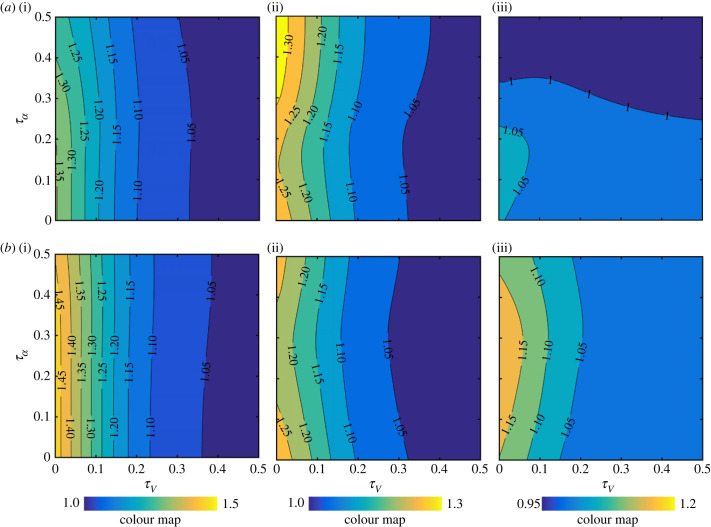
Effect of advancing wing rotation on wake capture characteristics. (*a*) Case of 5% advancement in rotation, and (*b*) case of 10% advancement in rotation. (i) Contour plots for $\left({\bar{L}}_{TR+WC}/{\bar{L}}_{TR}\right)$, (ii) contour plots for $\left({\bar{D}}_{TR+WC}/{\bar{D}}_{TR}\right)$, and (iii) contour plots for $\left(\left({\bar{L}}_{TR+WC}/{\bar{L}}_{TR})/({\bar{D}}_{TR+WC}/{\bar{D}}_{TR}\right)\right)$*.* In this demonstration, an effective wing aspect ratio of 3 and a switching gain value of 6 are used.

So, what are the best symmetric half-strokes kinematics waveforms that can exploit wake capture? The conducted analysis, based on the proposed model, provides a clear answer: the angle of attack should approach a constant value throughout half-strokes (i.e. $\tau_{\alpha }=0$). This will ensure a maximum lift gain together with best lift gain-to-drag penalty ratio. Additionally, the velocity should also approach a constant value throughout half-strokes (i.e. $\tau_{V}=0$), as this value will ensure a maximum lift gain. That said, as discussed in §3.1, advanced wing rotation kinematics could provide better lift gains and less drag penalties. As such, figure 11 demonstrates the effect of advancing the wing rotation by 5% (figure 11*a*) and 10% (figure 11*b*) of the flapping period.

Figure 11 leads to several useful insights. First, it confirms that as the wing rotation becomes more advanced, the aerodynamic advantage of wake capture becomes more evident. This is clear as the contour values of the lift ratio $\left({\bar{L}}_{TR+WC}/{\bar{L}}_{TR}\right)$ increase, the contour values of the drag ratio $\left({\bar{D}}_{TR+WC}/{\bar{D}}_{TR}\right)$ decrease, and the contour values of the lift gain-to-drag penalty ratio ($\left({\bar{L}}_{TR+WC}/{\bar{L}}_{TR})/({\bar{D}}_{TR+WC}/{\bar{D}}_{TR}\right)$) increase. Second, compared with the symmetric half-strokes wing kinematics, the aerodynamic benefit is more pronounced. This is most evident from the lift gain-to-drag penalty ratio contours which are now above unity. Third, as the wing pitching rotation advancement is increased, the contours of all the aerodynamic quantities become almost vertical. This is an interesting result showing that for advanced wing pitching rotation, the variation of the velocity profile is the primary controller of the aerodynamic performance. Finally, similar to the symmetric rotation case, as the translation velocity approaches a constant value throughout the half-stroke (i.e. *τ_V_* = 0), the wake capture aerodynamic benefit is increased.

## 4. Discussion

### 4.1. Importance of wake capture

One of the main motivators behind this work was to answer the question: to what extent is it essential to account for wake capture within theoretical quasi-steady aerodynamic models of hovering insect flight? This motivation stems from the fact that all available quasi-steady theoretical models ignore this mechanism, despite knowing of its existence, while still being capable of accurately estimating the net aerodynamic forces. There are two possible explanations: the first explanation comes from the results presented herein for how wake capture is affected by kinematics variation. It was shown in §3.2 that to maximize the lift benefit from wake capture the acceleration and deceleration durations should be minimized and hence the velocity approaches a constant value within half-strokes. Previous quasi-steady models have typically employed sinusoidal variations for velocity (or alternatively flapping angle) which is shown here to significantly minimize the aerodynamic influence from the wake capture mechanism. Note that a sinusoidal flapping profile provides a means to minimize the peak instantaneous mechanical power and torque. This is important in flapping flight as increased instantaneous peak power and torque lead to significant increase in the system weight. On the other hand, non-sinusoidal flapping profiles are costly in terms of peak instantaneous mechanical power and torque, which can offset the aerodynamic benefits. In the extreme case, near-constant flapping velocity kinematics (i.e. near-triangular flapping angle profiles) are possible in laboratory experimental rigs where weight is not an issue but do not read across into engineering [17] or biological [42] flight systems.

**Figure 12.**
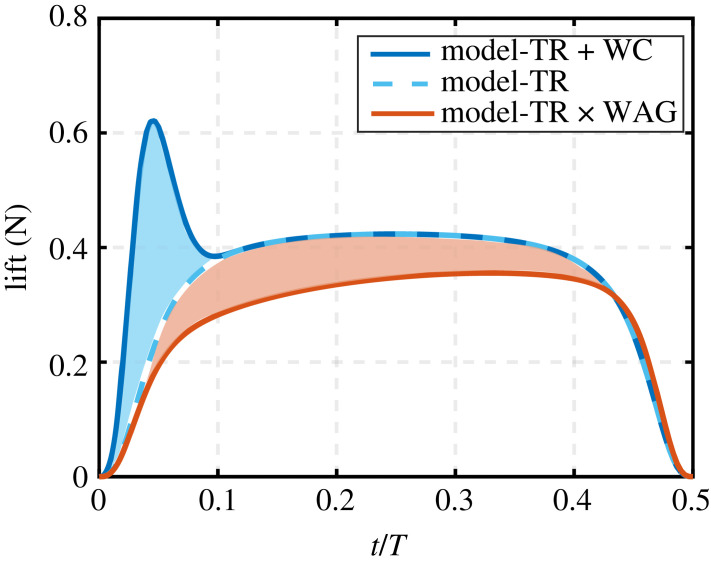
The influence of accounting for wake capture and Wagner effects on the reference fruit fly case with symmetric pitching rotation kinematics. Dark blue solid line represents the wake capture effect on top of the translation effect. Light blue dashed line represents translation effect only. Red Line represents translation effect coupled with the modified Wagner function.

A second explanation may be that there is another aerodynamic effect that cancels out the increase of lift caused by wake capture, and such possible mechanism may be the Wagner effect. The Wagner effect is an unsteady aerodynamic mechanism that delays lift generation, which happens because the aerodynamic forces are not produced instantly. This effect, however, has been neglected in the majority of available flapping wing models because it is inconsequential for three-dimensional flapping wings at low Reynolds numbers [5,7,8] (more details on the importance of Wagner effect could be found in [5]). In the following illustration, I considered the hypothetical case where both wake capture and Wagner effects coexist. Figure 12 shows such case for the fruit fly case (with simulation data sourced from table 1) employing symmetric pitching rotation kinematics. Figure 12 shows the translation effect modelled with the wake capture mechanism using the model developed in this work, as well as with the Wagner effect. Note that this work is neither meant to provide a detailed analysis nor an accurate predictive tool of the Wagner effect, but rather uses one of several possible simplified/low-order representations of this effect to demonstrate its potential contribution so as to support the discussion point made. As such, the translational lift modified by the Wagner effect was calculated using the equations

4.1 $$L(t)=\frac{1}{2}\rho {\left(\dot{\phi }(t)\right)}^{2}C_{L}(\alpha (t))\int_{0}^{R}c(r){\mathrm{Wag}}_{\mathrm{mod}}(r,t)r^{2}\;dr,$$

4.2 $${\mathrm{Wag}}_{\mathrm{mod}}(r,t)=\frac{\alpha (t)}{\alpha_{\infty }}\left(\frac{\tau (r,t)+2}{\tau (r,t)+4}\right)$$

4.3 $$\mathrm{and}\;\;\tau (r,t)=\frac{2\dot{\phi }(t)rt}{c(r)},$$

where *c* is the wing chord and *α*_∞_ is the steady state angle of attack value (45 degrees herein). Note that equation (4.2) is a modified form for the original Wagner function: the term in brackets is the well-known approximation of the Wagner function [43], whereas the term preceding the bracket is a modification for pitching non-impulsively starting wings as suggested by Babinsky *et al*. [44].

Clearly, it could be seen that while the wake capture contributes to an increase in the lift force, the opposite is achieved by the Wagner effect. For the specific fruit fly case shown in figure 12, the wake capture increases the lift by 11%, whereas the Wagner effect decreases the lift by 16%. For completeness, table 2 shows the contribution of wake capture and Wagner effects to lift for all the test cases previously presented in §3.1. Here, the wake capture contribution is calculated based on the model presented in this study, whereas the Wagner contribution is evaluated based on equations (4.1–4.3).

**Table 2.** Comparison between the wake capture and Wagner function contributions to lift for the different test cases considered in this study.

test case	wake capture contribution to lift (%)	Wagner function contribution to lift (%)
fruit fly, advanced kinematic case (figure 4*a*)	+13.8	−17.3
fruit fly, symmetric kinematic case (figure 4*b*)	+10.5	−16.2
fruit fly, delayed kinematic case (figure 4*c*)	−0.98	−15.6
hawkmoth, constant translation velocity over 76% of half-stroke kinematic case (figure 5*a*)	+24.9	−6.4
hawkmoth, constant translation velocity over 52% of half-stroke kinematic case (figure 5*b*)	+11.8	−7.5
hawkmoth, sinusoidal translation velocity kinematic case (figure 5*c*)	+4.7	−7.5
hawkmoth, constant rotation velocity over 76% of half-stroke kinematic case (figure 6*a*)	+14.4	−7.2
hawkmoth, constant rotation velocity over 52% of half-stroke kinematic case (figure 6*b*)	+11.8	−7.5
hawkmoth, sinusoidal rotation velocity kinematic case (figure 6*c*)	+7.7	−8.0

Looking at table 2, it is evident that kinematics plays a major role in determining the contributions of wake capture and Wagner effects. Moreover, there may be situations where the wake capture and Wagner effects almost cancel each other (such as in the discussed fruit fly case with symmetric kinematics). However, there are other cases where the contributions of both effects are vastly different (e.g. fruit fly delayed kinematics case and several hawkmoth cases). As such, the explanation that these two effects cancel each other does not hold in all cases. As discussed previously, existing quasi-steady models have shown good predictive capability without including either wake capture or Wagner effect. While there remains a possibility that these two effects may cancel each other out in some cases, the simpler explanation is that both these effects are small for cases of *practical* insect-scale flapping flight. In particular, the wake capture contribution is small because modelled flapping profiles are typically sinusoidal.

### 4.2. Limitations of the current model

The model proposed in this study, despite of its simplicity, proved to provide a convenient method to capture the force variations observed within the currently available experimental results in the literature. However, caution should be taken, as not all the input parameters to the proposed model could be evaluated based on a theoretical basis; hence, the model is in essence semi-empirical. This is because the value of (*t*/*T*)_cut_ was selected based on existing experimental and numerical data, and that the switching gain, *n*, was identified based on a best fit approach with available experiments. Unfortunately, there are currently only few experimental results that consider significantly different cases; hence, drawing general conclusions on the values for (*t*/*T*)_cut_ and *n* may be misleading. That said, the 0.1 value for (*t*/*T*)_cut_ seems to be reasonably accepted. This is based on the fact that all available experimental and numerical data to date were consistent in showing almost the same value for (*t*/*T*)_cut_ irrespective of the kinematic patterns used. For example, references [8,10,36] employed different kinematic patterns; however, the time period within the flapping cycle over which wing–wake interaction takes place was observed to be consistently the same around the value of 0.1.

The switching gain value is less straightforward. In the conducted comparison against the existing experimental data, it was shown that a switching gain value of four fits the fruit fly experimental results from Sane & Dickinson [8], whereas a value of eight fits the hawkmoth experimental measurements from Han *et al*. [32]. However, the important point to stress here is that for both cases, a single value of the switching gain was capable of modelling all the measurements for the different kinematic variations. This is remarkable and suggests that the switching gain is not dependent on the kinematics but most probably is influenced by the differences in measurement set-ups and/or differences in flow conditions. Indeed, the Reynolds number of the fruit fly experiment is of *O*(10^2^), whereas the Reynolds number of the hawkmoth experiment is of *O*(10^4^). Reynolds number is known to affect the shape of vortex structures in a profound way, which would in turn affect the amplitude of the wake capture force peaks. The recent study of Li & Nabawy [36] has shown that the wake capture effect becomes stronger as the Reynolds number increases over two-dimensional wings. Hence, the variation in the switching gain for different Reynolds numbers is not unexpected. In fact, as a preliminary starting point, it seems sensible to use an *n* value of four for Reynolds numbers of *O*(10^2^), six for Reynolds numbers of the *O*(10^3^), and eight for Reynolds numbers of the *O*(10^4^). Finally, the use of different switching gains to fit different experimental results should not be seen as a major concern as it was shown that the way the model input parameters affect the wake capture results is independent from the value of the switching gain. This is a very important outcome that means that the conclusions on optimum wake capture kinematics are general irrespective of the switching gain value. That said, there may be unique cases such as mosquitoes that lead to identifying different switching functions for their significantly different kinematics, flows and vortex structure patterns.

As a final comment on limitations, the model in the current study was compared against rigid wing cases. In fact, the literature on wake capture has mainly been limited to studying this mechanism for rigid wings. Clearly, real insect wings exhibit different degrees of flexibility, and such effect would be important to consider in future wake capture studies. However, the current state of the art on wake capture is somehow similar to the status of understanding of the other aerodynamic mechanisms two decades ago. At such time, the focus was mainly geared towards understanding the aerodynamics of rigid insect wings, and once that understanding was established, researchers started putting more focus on flexibility effects. I anticipate the same will happen with wake capture aerodynamics understanding, i.e. once enough understanding is developed for rigid wing cases, more studies will focus on the effect of flexibility on this unique aerodynamic mechanism. I also hope that the model presented in this work will be informative at such time. For example, with appropriate corrections, and if the spatial and temporal variations of the aerodynamic parameters within the model, e.g. the two-dimensional lift curve slope, are quantified for dynamically flexible wing cases, this may help in understanding some of the wake capture aerodynamic trends for flexible wings in a cost-effective fashion.

### 4.3. Final remarks

Currently, there is increased interest in proposing mathematical formulations that can deal with complex vortex-dominated flows using simple but accurate techniques. Good examples of such recent contributions can be found in [45,46] where a force and moment partitioning formulation based on the manipulation of kinematic and aerodynamic characteristics of the individual vortex structures is used to provide physically insightful understanding of the flow around immersed bodies. Similarly, the same philosophy was adopted here, where a novel simple analytical model has been developed for describing the complex wake capture aerodynamic mechanism, which can seamlessly be integrated into quasi-steady aerodynamic models of hovering insect flight. Despite its simplicity, the proposed model is yet reasonably accurate in predicting the force variations throughout the half-strokes, and this has been demonstrated through extensively comparing the model outputs with eight different experimental results from the literature. The proposed model is semi-empirical with only two parameters ((*t*/*T*)_cut_ and *n*) being determined based on best fit with experiments. That said, the exact value of the switching gain was shown to have no effect on the conclusions extracted on how the flapping kinematics can be varied to maximize the aerodynamic benefit of wake capture. In particular, the model confirmed that advanced wing pitching rotation would provide the best wake capture performance, i.e. highest lift gain and lowest drag penalty. Additionally, it was shown that the velocity profile within the flapping half-stroke is a main driver behind the observed lift and drag peaks. It was found that having a nearly constant velocity profile (or in other words having very short acceleration and deceleration phases within the half-stroke) would maximize the wake capture effect. This finding proved vital to understand why previous quasi-steady models that ignored wake capture still managed to predict the net aerodynamic forces with acceptable accuracy, as these models were simply employing wing kinematics that would minimize the exploitation of capturing the wake.

As a final comment on wake capture modelling, there is still a long way to go in terms of fully characterizing this challenging unsteady mechanism. However, in the absence of any explicit model to model wake capture, I believe that the current work provides a much-needed step to break the current stagnation status and take a step forward towards showing that this aerodynamic mechanism could still be modelled using theoretical means. More importantly, the work provided a framework that could capture the essential attributes of the problem, hence allowing for both useful insights as well as a future planning tool for further investigations.

## Data Availability

All data are contained in the paper.
